# Evaluation of enhanced home care support clinics regarding emergency home visits, hospitalization, and end-of-life care: a retrospective cohort study in a city of Japan

**DOI:** 10.1186/s12913-023-09088-1

**Published:** 2023-02-03

**Authors:** Yu Sun, Masao Iwagami, Nobuo Sakata, Tomoko Ito, Ryota Inokuchi, Jun Komiyama, Naoaki Kuroda, Nanako Tamiya

**Affiliations:** 1 Graduate School of Comprehensive Human Sciences, University of Tsukuba, Ibaraki, Japan; 2 Health Services Research and Development Center, University of Tsukuba, Ibaraki, Japan; 3 Department of Health Services Research, Faculty of Medicine, University of Tsukuba, Ibaraki, Japan; 4 Health Department, Tsukuba City, Ibaraki, Japan; 5 Department of Community Mental Health & Law, National Institute of Mental Health, National Center of Neurology and Psychiatry, Tokyo, Japan

**Keywords:** Home healthcare services, Emergency home visits, Terminal care

## Abstract

**Background:**

To meet the increasing demand for home healthcare in Japan as the population ages, home care support clinics/hospitals (HCSCs) and enhanced HCSCs were introduced in 2006 and 2012, respectively. This study aimed to evaluate whether enhanced HCSCs fulfilled the expected role in home healthcare.

**Methods:**

We conducted a retrospective cohort study using linked medical and long-term care claims data from a municipality in Japan. Participants were ≥ 65 years of age, had newly started regular home visits between July 2014 and March 2018, and used either conventional or enhanced HCSCs. Patients were followed up for one year after they started regular home visits or until the month following the end of the regular home visits if they ended within one year. The outcome measures were (i) emergency home visits at all hours and on nights and holidays at least once, respectively, (ii) hospitalization at least once, and (iii) end-of-life care, which was evaluated based on the place of death and whether a physician was present at the time of in-home death. Multivariable logistic regression analyses were conducted for the outcomes of emergency home visits and hospitalizations.

**Results:**

The analysis included 802 patients, including 405 patients in enhanced HCSCs and 397 patients in conventional HCSCs. Enhanced HCSCs had more emergency home visits at all hours than conventional HCSCs (65.7% vs. 49.1%; adjusted odds ratio 1.70, 95% CI [1.26–2.28]), more emergency home visits on nights and holidays (33.6% vs. 16.7%; 2.20 [1.55–3.13]), and fewer hospitalizations (21.5% vs. 32.2%; 0.55 [0.39–0.76]). During the follow-up period, 229 patients (152 patients in enhanced HCSCs and 77 patients in HCSCs) died. Deaths at home were significantly more common in enhanced HCSCs than in conventional HCSCs (80.9% vs. 64.9%; *p* < .001), and physician-attended deaths among those who died at home were also significantly more common in enhanced HCSCs (99.2% vs. 78.0%; *p* < .001).

**Conclusions:**

This study confirms that enhanced HCSCs are more likely to be able to handle emergency home visits and end-of-life care at home, which are important medical functions in home healthcare. Further promotion of enhanced HCSCs would be advantageous.

**Supplementary Information:**

The online version contains supplementary material available at 10.1186/s12913-023-09088-1.

## Background

Over the past few decades, the organization of primary healthcare has changed in many countries [1]. Many Western countries are shifting from single-handed general practitioners (GPs) to group practice [2]. These changes are due to changes in GPs’ attitudes, increasing patient demand for after-hour care, and regional shortages of primary care physicians [3, 4].

In Japan, where the population is aging the fastest in the world [5], promoting home healthcare to support patients through their end-of-life stage has become an urgent issue [6]. Home healthcare in Japan entails physicians making regular home visits to diagnose and monitor medical conditions, as well as prescribing medications. The Ministry of Health, Labour, and Welfare (MHLW) identified four medical functions as necessary for home healthcare: discharge assistance through collaboration with hospital staff, daily life support, which includes assistance for family caregivers and palliative care for patients, emergency home visits for acute illnesses or exacerbations of chronic conditions, and end-of-life care at home [7]. Home healthcare is initiated when a patient cannot visit the clinic/hospital they originally visited as an outpatient, or when another medical facility decided that home healthcare is required and refers the patient to a clinic/hospital that provides home healthcare. Regular home visits were often made once or twice a month. Patients who receive physician home visits often use the nursing care visits and home help services provided by various care facilities [8].

The MHLW introduced home care support clinics and hospitals (HCSCs) in 2006 [9]. HCSCs have a system in place that enables 24-h home visiting care at the patient’s request (a conventional requirement of all HCSCs). In addition, to provide higher-quality home healthcare, especially for emergency home visits and end-of-life care, enhanced HCSCs were introduced in 2012 [9]. Enhanced HCSCs should meet all of the following requirements added to the conventional ones: three or more full-time doctors appointed, ten or more cases of physician-led emergency home visit in the past year, and four or more cases of providing end-of-life care at home in the past year (This is the requirement after 2014) [9, 10]. If an HCSC meets the enhanced requirements, it can apply to become an enhanced HCSC and obtain a higher fee than that of a conventional HCSC [9, 10]. If a patient started receiving home healthcare from an HCSC, that HCSC would cover all the patient's primary care.

Several previous studies evaluated the outcomes of the medical function of HCSCs (which included both the conventional and enhanced versions). A previous study showed that the rate of readmission within 30 days of discharge was lower in patients who received HCSCs [11]. According to other ecological research, municipalities with higher rates of home deaths were more likely to have greater resources for HCSCs [12, 13]. These studies suggested that HCSCs performed more roles expected in home healthcare than general clinics, yet the difference between conventional and enhanced HCSCs was unclear. Another study revealed that patients who used enhanced HCSCs with beds had a high utilization of home-based end-of-life care and relatively low medical institutional deaths. This indicated that enhanced HCSCs performed their expected role more in end-of-life care [14]. However, differences in other medical functions, such as emergency home visits, are unclear.

Therefore, we aimed to compare the outcomes of medical functions expected in home healthcare between enhanced HCSCs and conventional HCSCs focusing on emergency home visit, hospitalization, and end-of-life care, and examine whether enhanced HCSCs had fulfilled the expected role.

## Methods

### Data source

We obtained linked data on medical and long-term care insurance claims from the municipal government of Tsukuba City in Ibaraki Prefecture, Japan. Tsukuba City had a population of approximately 212,000, with approximately 41,000 (19.3%) people aged ≥ 65 years in 2015 [15]. For this study, data was selected from April 2014 to March 2019.

Medical claims data included data from individuals with National Health Insurance for the self-employed and retirees under 75 years and Late-stage medical care system for older adults aged 75 and older. Data from individuals with other health insurance credentials (e.g., insurance for corporate employees) were not included [16, 17]. Medical insurance claims records included the monthly information of diagnoses, medical procedures, and prescriptions. The recorded diagnoses were based on the original Japanese disease codes linked to the International Classification of Diseases Tenth Revision (ICD-10) codes [18].

Under the statutory long-term care insurance system, older people who need living assistance can receive care services based on seven levels of the certificate of need for long-term care: Support 1 (lowest disability) to 2 and Care 1 to 5 (highest disability) [19]. All Japanese citizens aged ≥ 65 years and individuals aged 40–64 years whose need for care is derived from age-related diseases, such as stroke, cancer, and rheumatoid arthritis, are eligible for benefits. The long-term care need level is a nationally standardized certification assessed based on a person’s physical and cognitive functioning [20]. Long-term care insurance claims data contain information on care need levels and services used by all residents receiving long-term care services.

The municipal government linked medical and long-term claims data using personally identifiable information. In the data we received, anonymized ID numbers were assigned to individuals in both the medical and long-term care insurance claim datasets.

### Study design and population

This was a retrospective, cohort study. Individuals who had newly started regular home visits between July 2014 and March 2018 in Tsukuba City and those who used either enhanced HCSCs or conventional HCSCs were included (*n* = 1,006). Individuals who did not receive regular home visits between April and June 2014 in Tsukuba City were considered newly enrolled. First, we excluded people whose medical and long-term care claims data could not be linked (*n* = 193). The long-term care claims data was based on data at the end of the fiscal year. Therefore, we were unable to link to a person who had died or moved before the end of any fiscal year. Next, we excluded individuals aged < 65 years at the time they started regular home visits (*n* = 11). The age of 65 years was chosen as the lowest limit because (i) all people aged ≥ 65 years were eligible for long-term care insurance benefits and (ii) the vast majority (over 95%) of regular home visits were conducted for this age group [7].

### Exposure and outcome

Exposure was the type of medical facility that provided home healthcare: enhanced or conventional HCSCs. It was identified from the medical insurance claims data from the month when regular home visits were initiated.

We evaluated three outcomes of the medical functions for home healthcare: (i) emergency home visit, (ii) hospitalization, and (iii) end-of-life care. Patients were followed up for one year after the start of the regular home visit or until the month following the end of the regular home visit if they ended within one year. The presence/absence of (in the main analysis) and the number of (in the sensitivity analysis) emergency home visits and hospitalizations during the follow-up period were identified. We extracted emergency home visits at all hours and on nights and holidays, respectively. Emergency home visits on nights and holidays were defined as between 6 PM and 8 AM on weekdays, weekends, and public holidays. Hospitalization of ≤ 2 days was excluded due to the possibility of hospitalization for planned examination, which would affect the evaluation of hospitalization that required treatment. For the third outcome (end-of-life care), we categorized those who died during the follow-up period by place of death (home or hospital), and if they died at home, we explored whether a physician was present at the time of death, based on the surcharges for end-of-life care at home. These were calculated for medical care given before and after death, including being present at the time of death. All variables were extracted from the medical insurance claims data.

### Covariates

The following covariates were extracted as potential confounding factors: age (categorized as 65–74, 75–84, 85–94, or ≥ 95 years), sex, long-term care need levels (classified as support levels 1–2, care need levels 1–3, and care need levels 4–5), Charlson Comorbidity Index (CCI), use of home nursing care services, and use of home oxygen therapy [21]. The CCI was initially developed as a disease-weighted score to predict mortality. However, it is also known to represent the burden of multimorbidity status [22, 23]. We used the 2011 updated and reweighted version of the original CCI scores, which was validated using a Japanese national administrative dataset [24]. The CCI scores were calculated based on the following diseases: congestive heart failure, dementia, chronic pulmonary disease, rheumatological disease, mild liver disease, diabetes mellitus, hemiplegia and paraplegia, renal disease, cancer, moderate/severe liver disease, metastatic solid tumors, and HIV/acquired immunodeficiency syndrome. We identified these diseases from medical insurance claims data during the three months before the start of regular home visits. In this study, the CCI was categorized into six groups (0, 1, 2, 3, 4, and ≥ 5) [24, 25]. The use of home nursing care services was identified from home nursing orders by physicians six months before the start of the regular home visit or from codes for home nursing care services in medical and long-term insurance claims data for the month of the start of the regular home visits. The use of home oxygen therapy was defined based on medical claims data for the month the regular home visits began.

### Statistical analysis

First, we obtained the summary statistics for each variable, according to the type of medical facility. In addition to the covariates, we also examined the prevalence of common diseases included in the CCI (diseases with a prevalence of 10% or more), follow-up period, and number of drug types prescribed during the three months before the start of regular home visits. Drugs used during hospitalization or prescribed for less than 14 days were not included due to the possibility of temporary medications.

Proportions were compared using chi-squared (χ^2^) tests and Student’s t-tests for binary and continuous variables, respectively. We calculated the crude proportion of (i) the presence of (i.e., at least one) emergency home visits at all hours and on nights and holidays at least once, and (ii) the presence of (i.e., at least one) hospitalization by the type of medical facility. Subsequently, we performed univariable and multivariable logistic regression analyses, in which the outcome was the presence or absence of an emergency home visit at all hours and on nights and holidays, (i.e., at least one each), as well as hospitalization (i.e., at least one hospitalization) during the follow-up period, and the exposure was the type of home care facility, adjusted for the aforementioned covariates (i.e., age, sex, long-term care need levels, CCI, use of home nursing care service, and use of home oxygen therapy). We also illustrated a scatter plot of the average number of home visits per month versus the number of hospitalizations during each observation period. We calculated the average number of emergency home visits per month by dividing the total number of emergency home visits by the number of months that each person received regular home visits.

Outcomes of end-of-life care were examined in those who died during the follow-up period. We compared the place of death (home, hospital, or unknown) and whether a physician was present at the time of death among patients who died at home between enhanced and conventional HCSCs using a χ^2^ test and Fisher’s exact test.

We performed several sensitivity analyses to examine the robustness of our analysis. First, we performed a multivariable negative binomial regression for the number of emergency home visits and hospitalizations. Second, we restricted the analysis to the outcomes of emergency home visits and hospitalizations for patients who had not died during the follow-up period. This was because we were concerned that emergency home visits to confirm death may be included in the main analysis, and the ability to make emergency home visits for a sudden deterioration in the patient’s condition could not be properly evaluated. Finally, we conducted a sensitivity analysis limited to patients without a diagnosis of cancer because cancer patients are generally more likely to require emergency home visits or hospitalization due to the deterioration of their condition [21, 26].

All analyses were conducted using STATA version 15 (Stata Corp., Texas, USA). Statistical significance was set at *p* < 0.05.

## Results

**Fig. 1:**
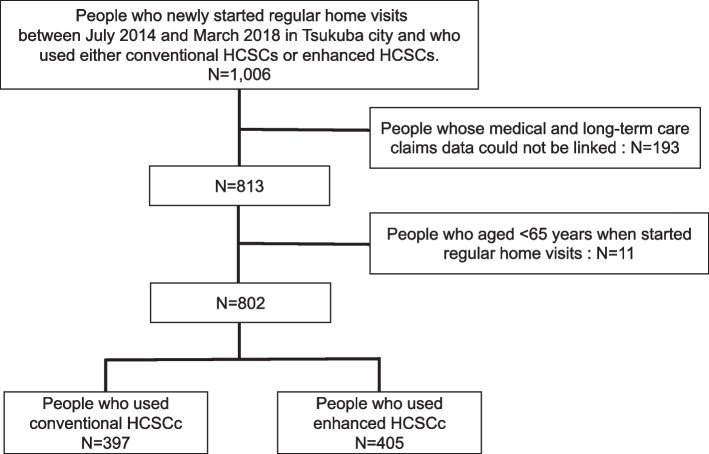
Flow chart of the study participants’ selection. HCSCs = home care support clinics/hospitals

After applying the exclusion criteria, 802 patients, including 405 patients in enhanced HCSCs and 397 patients in conventional HCSCs, were ultimately included in the analysis (Fig. 1).

Table 1 shows the characteristics of the participants according to the type of medical facility. Compared with people who used conventional HCSCs, those who used enhanced HCSCs were more likely to have high care need level certification and used home nursing care services and home oxygen therapy. Meanwhile, those who used conventional HCSCs were more likely to have dementia. The follow-up time was significantly shorter in enhanced HCSCs.

**Table 1:** Characteristics of the patients in conventional HCSCs and enhanced HCSCs

	Conventional HCSCs	Enhanced HCSCs	
	*N* = 397	*N* = 405	
	n (%)	n (%)	*p*-value
**Mean age, years (SD)**	84.7 (7.5)	85.3 (7.7)	0.327
**Age category (years)**			0.061
65−74	39 (9.8)	44 (10.9)	
75−84	142 (35.8)	128 (31.6)	
85–94	192 (48.4)	188 (46.4)	
≥ 95	24 (6.1)	45 (11.1)	
**Sex: male**	145 (36.5)	151 (37.3)	0.824
**Long-term care need levels**			0.001
Care support levels 1–2	16 (4.0)	11 (2.7)	
Care need levels 1–3	249 (62.7)	206 (50.9)	
Care need levels 4–5	132 (33.3)	188 (46.4)	
**Charlson Comorbidity Index (CCI)**			0.335
0	37 (9.3)	41 (10.1)	
1	20 (5.0)	35 (8.6)	
2	113 (28.5)	106 (26.2)	
3	65 (16.4)	55 (13.6)	
4	48 (12.1)	45 (11.1)	
≥ 5	114 (28.7)	123 (30.4)	
**Common diseases included in the CCI**			
Congestive heart failure	138 (34.8)	147 (36.3)	0.650
Dementia	221 (55.7)	179 (44.2)	0.001
Chronic pulmonary disease	109 (26.5)	114 (28.2)	0.827
Mild liver disease	60 (15.1)	83 (15.6)	0.862
Diabetes mellitus	127 (31.2)	141 (34.8)	0.396
Cancer	77 (19.4)	93 (23.0)	0.216
**Use of home nursing care services**	158 (39.8)	211 (52.1)	< 0.001
**Use of home oxygen therapy**	14 (3.5)	34 (8.4)	0.004
**Number of months followed (mean, SD)**	8.6 (4.6)	7.3 (5.1)	<0.001
**Number of drug types prescribed (mean, SD)**	6.8 (3.5)	6.7 (3.4)	0.700

We conducted Student’s t-test for continuous variables and χ2 test for categorical variables

*Abbreviations*: *SD* Standard deviation, *HCSCs* Home care support clinics/hospitals, *CCI* Charlson Comorbidity Index

Enhanced HCSCs had more emergency home visits at all hours than conventional HCSCs (65.7% vs. 49.1%), more emergency home visits on nights and holidays (33.6% vs. 16.7%), and fewer hospitalizations (21.5% vs. 32.2%).

**Fig. 2:**
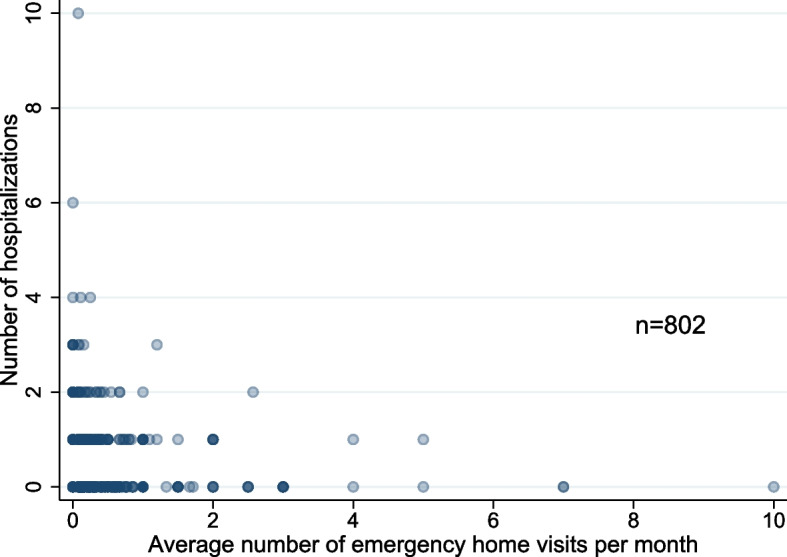
Scatter plots between the average number of emergency home visits per months and hospitalizations

Table 2 shows the results of the univariable and multivariable logistic regression analyses for the outcomes of emergency home visits and hospitalizations. Compared with conventional HCSCs, the adjusted odds ratio (OR) and 95% confidence intervals (CI) for enhanced HCSCs were 1.70 [1.26–2.28] and 2.20 [1.55–3.13] for emergency home visits at all hours and emergency home visits on nights and holidays, respectively. The adjusted OR [95% CI] for the hospitalization was 0.55 [0.39–0.76] for enhanced HCSCs. The scatter plot between the average number of home visits per month and number of hospitalizations showed that patients who received frequent home visits tended to have fewer hospitalizations (Fig. 2).

**Table 2:** Univariable and multivariable logistic regression analyses for the outcome of emergency house calls and hospitalizations

		Univariable		Multivariable	
	Incidence	OR (95% CI)	*p*-value	aOR* (95%CI)	*p*-value
**Emergency home visits at all hours at least once**					
Conventional HCSCs	195/397 (49.1)	(reference)	< 0.001	(reference)	< 0.001
Enhanced HCSCs	266/405 (65.7)	1.98 (1.49–2.63)		1.70 (1.26–2.28)	
**Emergency home visits on nights and holidays at least once**					
Conventional HCSCs	66/397 (16.7)	(reference)	< 0.001	(reference)	< 0.001
Enhanced HCSCs	136/405 (33.6)	2.54 (1.81–3.55)		2.20 (1.55–3.13)	
**Hospitalizations at least once**					
Conventional HCSCs	128/397 (32.2)	(reference)	0.001	(reference)	< 0.001
Enhanced HCSCs	87/405 (21.5)	0.57 (0.42–0.79)		0.55 (0.39–0.76)	

aOR* was adjusted for age, sex, long-term care need levels, Charlson Comorbidity Index, and the use of home nursing care services and home oxygen therapy

*Abbreviations*: *aOR* Adjusted odds ratio, *CI* Confidence interval, *HCSCs* Home care support clinics/hospitals

During the follow-up period, 229 patients died, including 152 patients with enhanced HCSCs and 77 patients with conventional HCSCs. Table 3 shows the number and proportion of the places of death and presence or absence of a physician at the time of in-home death in each type of medical facility. All in-home deaths and physician-attended in-home deaths were significantly more common in enhanced HCSCs, whereas in-home death without the presence of a physician and death in the hospital were more common in conventional HCSCs.

**Table 3:** Place of death and presence of a physician at time of death in each facility

Place of death			
	Conventional HCSCs	Enhanced HCSCs	
	*N* = 77	*N* = 152	
	n (%)	n (%)	*p*-value
Death at home	50 (64.9)	123 (80.9)	< 0.001
Death in hospital	24 (28.6)	14 (9.2)	< 0.001
Unknown	5 (6.5)	15 (9.9)	< 0.001
Presence of a physician at the time of in-home death			
	conventional HCSCs	enhanced HCSCs	
	*N* = 50	*N* = 123	
	n (%)	n (%)	*p*-value
With the presence of physician at the time of death	39 (78.0)	122 (99.2)	< 0.001
Without the presence of physician at the time of death	11 (22.0)	1 (0.8)	< 0.001

We conducted χ2 test and Fisher’s exact test

*Abbreviations*: *HCSCs* Home care support clinics/hospitals

In the sensitivity analysis, a multivariable negative binomial regression analysis revealed that enhanced HCSCs had significantly fewer emergency home visits and hospitalizations (Supplementary Table 1). The analysis restricted to patients who had not died during the follow-up period showed no significant difference in follow-up time between the two groups. Nevertheless, the results were similar to the main analysis, although the significance of emergency home visits at all hours was marginal (Supplementary Table 2). The results of the sensitivity analysis of patients without a diagnosis of cancer were consistent with those of the main analysis (Supplementary Table 3).

## Discussion

The results showed that enhanced HCSCs had more emergency home visits and fewer hospitalizations than conventional HCSCs. In addition, enhanced HCSCs provided more end-of-life care at home with a physician present at the time of death. These findings suggest that enhanced HCSCs take on more roles in home healthcare, such as emergency home visits and end-of-life care at home, than conventional HCSCs.

We found that enhanced HCSCs had more emergency home visits and fewer hospitalizations. A previous study that evaluated readmissions within 30 days of discharge showed that HCSCs had fewer readmissions [11]. However, the mechanism was unknown. Our results showed that patients with more frequent emergency home visits tended to be hospitalized less, suggesting that hospitalization may be reduced by making emergency home visits with enhanced HCSCs. Notably, the point estimate of the odds ratios was higher for emergency home visits on nights and holidays than for all hours. This may be since enhanced HCSCs require at least three full-time physicians, and having a large number of physicians may facilitate making emergency home visits, particularly on nights and holidays. Enhanced HCSCs may tend to have a policy of providing end-of-life care at home instead of hospitalization when the patient’s condition deteriorates, leading to fewer hospitalizations. Conventional and enhanced HCSCs can obtain additional fees if they provide end-of-life care at home. However, the results of the analysis that were restricted to patients who had not died were similar. Therefore, even putting aside the policy of end-of-life care for those who are close to death, enhanced HCSCs are more likely to make emergency home visits rather than admitting the patient to the hospital for a rapid worsening of their condition.

Our study revealed that enhanced HCSCs resulted in more deaths at home than conventional HCSCs, which was consistent with the results of a previous study [14]. Previous studies reported that the in-home death was associated with better quality of death and lower caregiver burden than death in palliative care units or hospitals [27, 28]. While more than half of the Japanese people stated that they would prefer to die at home [29], only approximately 13% achieved this goal in 2017 [30]. To fill this gap, measures to increase enhanced HCSCs would be required.

In addition, physicians were more likely to be present at the time of death in patients with enhanced HCSCs. Physician presence at the time of death (including medical care given before and after death) may lead to family caregiver satisfaction and a better quality of death. Notably, 22% of in-home deaths were not attended by physicians at the time of death in conventional HCSCs, suggesting that low staffing levels in this condition made it difficult for physicians to attend at the time of death.

In the current study, 152/405 (37.5%) in enhanced HCSCs and 77/397 (19.4%) in conventional HCSCs died during the follow up, which showed a higher mortality in enhanced HCSCs. Enhanced HCSCs were reported to have a higher proportion of facilities that could handle patients with more complex medical requirements, such as palliative care for patients with terminal cancer [31, 32]. Therefore, patients who were more seriously ill may be referred to the enhanced HCSCs, and differences in patient characteristics may have an impact on mortality rates.

Our findings suggest that group practice improved the outcomes expected in home healthcare, which is consistent with previous studies in foreign countries [2, 33, 34]. Over the past few decades, group practice has been adopted in many countries as an important part of the process of the primary healthcare promotion and currently represents the basis of modern healthcare systems [2]. These studies showed that group practice had many benefits for patients and physicians, such as improved patient satisfaction, quality of care, and physicians’ quality of life [2, 33, 34]. Many Western countries have established GP cooperatives for after-hours medical care [3, 4, 35–37]. In this system, GPs work in a nonprofit organization and take turns on duty after-hours for the patient population of all participating GPs [35]. The quality of care at GP cooperatives has proven to be good, and both patients and healthcare professionals reported being satisfied [3, 37]. A previous report showed that more than 70% of physicians at HCSCs (including both conventional and enhanced versions) felt burdened by the 24-h system, but physicians at three or more full-time doctors appointed HCSCs felt less burdened [38]. Therefore, large-scale group practices for after-hours medical care may help reduce the burden on primary care physicians in Japan.

Although the number of HCSCs is increasing, enhanced HCSCs accounted for only 24% of the total HCSCs in 2018 in Japan [10]. Most conventional HCSCs are single-handed practices and have difficulties providing three or more full-time doctors [39]. In such conditions, to further expand the role of home healthcare that enhanced HCSCs play, new measures may need to be considered, such as delegating home visits to another healthcare professional [40, 41] or establishing organizational models for after-hours medical care [35], as in other countries.

This study had several limitations. First, we adjusted for confounders that could have been associated with the type of medical facilities and outcomes in the analyses; however, the observed association could have been influenced by residual confounding factors, such as detailed data on health status, living status, and socioeconomic factors. Second, although the current study used data from a relatively large city in Japan, the sample size and range of outcomes did not allow us to adjust for a wide range of diseases. Therefore, we used the CCI to roughly estimate the influence of comorbidity burden and adjusted for it in the association between the type of medical facilities and outcomes. Third, although in-home death was influenced by the patient’s wishes, family situation, and advance care planning, we were unable to identify these factors. Fourth, since the requirements for enhanced HCSCs included emergency home visits and end-of-life care, supplier-induced demand may affect outcomes. However, our study also showed that hospitalization was lower in patients with enhanced HCSCs. Therefore, we consider that emergency home visits of enhanced HCSCs may help prevent hospitalization. Finally, due to the limited geographical location examined, it is uncertain how our findings can be applied to other areas; wide-ranging findings would have clear policy implications.

## Conclusions

In this population-based study using merged medical and long-term insurance claims data in Japan, we found that enhanced HCSCs had more emergency home visits, fewer hospitalizations, and more in-home deaths in the presence of physicians. These findings suggest that further measures to promote enhanced HCSCs should be reconsidered, and further studies are necessary to examine the generalizability of these findings.

## Supplementary Information

**Additional file 1:** **Supplementary Table 1. **Multivariable negative binomial regression analyses for the number of emergency home visits and hospitalizations. **Supplementary Table 2. **Multivariable logistic regression analyses for emergency home visits and hospitalizations, excluding the dead during follow-up. **Supplementary Table 3. **Multivariable logistic regression analyses for emergency home visits and hospitalizations for patients without cancer diagnosis.

## Data Availability

The data that support the findings of this study are available from Tsukuba city but restrictions apply to the availability of these data, which were used under license for the current study, and so are not publicly available. Data are however available from the authors upon reasonable request and with permission of Tsukuba city.

## Abbreviations

HCSCs: Home care support clinics/hospitals
GP: General practitioner
MHLW: Ministry of Health, Labour and Welfare
CCI: Charlson Comorbidity Index
OR: Odds ratio
CI: Confidence interval
