# Supplementary material for: Evaluation of enhanced home care support clinics regarding emergency home visits, hospitalization, and end-of-life care: a retrospective cohort study in a city of Japan
Source: BMC Health Serv Res. 2023 Feb 3;23:115. doi: 10.1186/s12913-023-09088-1 (PMC9898920; doi:10.1186/s12913-023-09088-1)
Supplement: Supplementary file 1 — Additional file 1: Supplementary Table 1. Multivariable negative binomial regression analyses for the number of emergency home visits and hospitalizations. Supplementary Table 2. Multivariable logistic regression analyses for emergency home visits and hospitalizations, excluding the dead during follow-up. Supplementary Table 3. Multivariable logistic regression analyses for emergency home visits and hospitalizations for patients without cancer diagnosis. [file 12913_2023_9088_MOESM1_ESM.docx]

**Supplementary Table 1. Multivariable negative binomial regression analyses for the number of emergency home visits and hospitalizations.**

|  | Median (Range) | Coefficient* (95%CI) | p-value |
| --- | --- | --- | --- |
| **Number of emergency home visits at all hours** | | | |
| Conventional HCSCs | 0 (0–18) | (reference) | 0.003 |
| Enhanced HCSCs | 1 (0–12) | 0.28 (0.10–0.47) |  |
| **Number of emergency home visits on nights and holidays** | | | |
| Conventional HCSCs | 0 (0–5) | (reference) | 0.001 |
| Enhanced HCSCs | 0 (0–5) | 0.51 (0.22–0.80) |  |
| **Number of hospitalizations** | | | |
| Conventional HCSCs | 0 (0–6) | (reference) | 0.012 |
| Enhanced HCSCs | 0 (0–10) | -0.36 (-0.63–-0.08) |  |

*Note:* Abbreviations: CI, confidence interval; HCSCs, home care support clinics/hospitals.

Coefficient * was adjusted for age, sex, long-term care need levels, Charlson Comorbidity Index, and the use of home nursing care services and home oxygen therapy.

.

**Supplementary Table 2. Multivariable logistic regression analyses for emergency home visits and hospitalizations, excluding the dead during follow-up.**

|  | Incidence (%) | aOR* (95%CI) | p-value |
| --- | --- | --- | --- |
| **Emergency home visits at all hours at least once** | | | |
| Conventional HCSCs | 135/320 (42.2) | (reference) | 0.058 |
| Enhanced HCSCs | 133/253 (52.6) | 1.40 (0.99–1.98) |  |
| **Emergency home visits on nights and holidays at least once** | | | |
| Conventional HCSCs | 29/320 (9.1) | (reference) | 0.008 |
| Enhanced HCSCs | 51/253 (20.2) | 2.03 (1.20–3.43) |  |
| **Hospitalizations at least once** |  |  |  |
| Conventional HCSCs | 101/320 (31.6) | (reference) | 0.025 |
| Enhanced HCSCs | 66/253 (26.1) | 0.63 (0.43–0.94) |  |

*Note:*Abbreviations: aOR, adjusted odds ratio; CI, confidence interval; HCSCs, home care support clinics/hospitals.

aOR* was adjusted for age, sex, long-term care need levels, Charlson Comorbidity Index, and the use of home nursing care services and home oxygen therapy.

**Supplementary Table 3. Multivariable logistic regression analyses for emergency home visits and hospitalizations for patients without cancer diagnosis.**

|  | Incidence (%) | aOR* (95%CI) | p-value |
| --- | --- | --- | --- |
| **Emergency home visits at all hours at least once** | | | |
| Conventional HCSCs | 159/317 (50.2) | (reference) | 0.005 |
| Enhanced HCSCs | 197/305 (64.6) | 1.62 (1.15–2.27) |  |
| **Emergency home visits on nights and holidays at least once** | | | |
| Conventional HCSCs | 47/317 (14.8) | (reference) | <0.001 |
| Enhanced HCSCs | 92/305 (30.2) | 2.21 (1.46–3.34) |  |
| **Hospitalizations at least once** | | | |
| Conventional HCSCs | 98/317 (30.9) | (reference) | <0.001 |
| Enhanced HCSCs | 62/305 (20.3) | 0.52 (0.35–0.77) |  |

*Note:*Abbreviations: aOR, adjusted odds ratio; CI, confidence interval; HCSCs, home care support clinics/hospitals.

aOR* was adjusted for age, sex, long-term care need levels, Charlson Comorbidity Index, and the use of home nursing care services and home oxygen therapy.

.
